# Pulmonary Metastasis of Anal Squamous Cell Carcinoma Nine Years Following Treatment

**DOI:** 10.7759/cureus.98238

**Published:** 2025-12-01

**Authors:** Maxwell S Madani, Anushka Deogaonkar, Athanasios S Naum, Robert Gordon, Marie L Borum

**Affiliations:** 1 Gastroenterology and Liver Diseases, George Washington University School of Medicine and Health Sciences, Washington, DC, USA; 2 Internal Medicine, George Washington University School of Medicine and Health Sciences, Washington, DC, USA

**Keywords:** distant recurrence, hiv, immunosuppressed, scca, squamous cell carcinoma of anus

## Abstract

Squamous cell carcinoma of the anus (SCCA), the predominant histologic subtype of anal cancer, is associated with human papillomavirus (HPV) infection in over 90% of cases. The risk of SCCA is markedly elevated in individuals with HIV, particularly those experiencing advanced or prolonged immunosuppression. Most local recurrences occur within three to five years of initial treatment, while distant metastases develop in 10-20% of patients following curative therapy. At diagnosis, distant metastases are identified in only 5-8% of cases. Prognosis varies significantly by stage, with a five-year overall survival of approximately 78% in localized disease and 19% in cases with distant metastasis.

We report a rare case of delayed pulmonary metastasis of SCCA in a 51-year-old man with HIV and a history of inconsistent adherence to highly active antiretroviral therapy (HAART). The patient had undergone definitive chemoradiation for anal squamous cell carcinoma nine years earlier, with no evidence of recurrence on routine surveillance. He presented to the emergency department with a four-day history of night sweats, chills, chest pain, abdominal discomfort, hematochezia, and unintentional weight loss. Computed tomography (CT) angiography revealed a 2.7 cm spiculated nodule in the left lower lobe of the lung, initially raising concern for a primary pulmonary malignancy. However, CT-guided biopsy demonstrated squamous cell carcinoma positive for P40 and P16, consistent with HPV-associated disease originating from the anorectal region. Colonoscopy revealed no signs of local recurrence.

This case highlights an exceptionally prolonged interval between curative treatment and metastasis of SCCA and raises important questions about the role of intermittent HAART adherence in long-term oncologic outcomes. It also underscores the need for clinicians to maintain a high index of suspicion for metastatic spread or recurrence of prior cancers in immunocompromised patients, even in the absence of local symptoms. Additional research is necessary to improve long-term surveillance strategies and mitigate recurrence risk in this vulnerable population.

## Introduction

Squamous cell carcinoma of the anus (SCCA), the most common histologic subtype of anal cancer, is driven by human papillomavirus (HPV) infection in over 90% of cases [1,2]. Additional risk factors include HIV infection, age, sex, ethnicity, reproductive history, healthcare access, environmental and geographic conditions, genetic predisposition, and other medical conditions that may affect immune function [3]. Individuals with HIV, particularly those with advanced immunosuppression, are at markedly increased risk of developing SCCA [4]. The incidence of SCCA has risen at approximately 2.7% annually over the past decade [5].

Most local recurrences occur within three to five years of diagnosis, and the risk of distant metastasis following curative local treatment is 10-20%. At presentation, distant metastases are observed in only 5-8% of cases [6,7]. The most common metastatic sites include the liver and lungs, followed by extra-pelvic lymph nodes, peritoneum, and bone [4]. Five-year overall survival for localized disease is ~78%, compared with 19% in patients with distant metastasis [8].

Late metastasis beyond five years is rare. A small number of published cases have described delayed pulmonary metastasis after seven to eight years [3]. To our knowledge, a nine-year disease-free interval to metastasis represents one of the longest reported intervals in the literature, highlighting the clinical significance of this case.

## Case presentation

A 43-year-old man with HIV initially presented to the emergency department with progressive rectal pain and drainage of several months’ duration. He had discontinued highly active antiretroviral therapy (HAART) due to psychosocial stress but later resumed therapy (ritonavir, darunavir, dolutegravir, and tenofovir/emtricitabine).

Examination findings

A 3×2 cm friable, fixed mass was palpated in the posterior midline on digital rectal exam. No externalized mass or palpable peripheral lymphadenopathy was noted. The patient had mild weight loss at the time but no systemic symptoms. Laboratory results showed normal lactate dehydrogenase (LDH) and normal inflammatory markers (C-reactive protein (CRP) and erythrocyte sedimentation rate (ESR)).

Imaging

**Figure 1:**
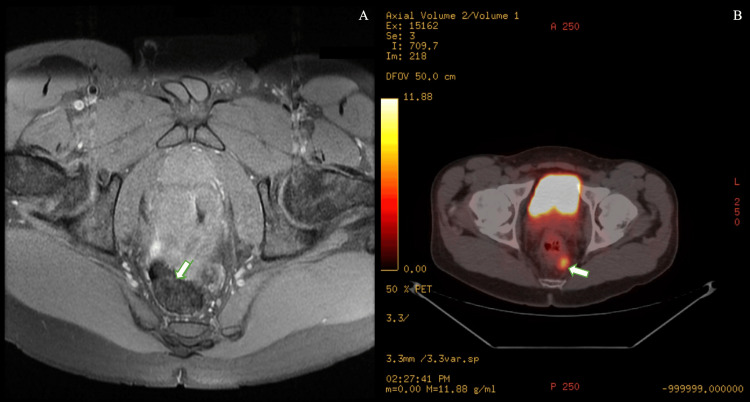
A) A 1.7 cm enhancing soft tissue lesion in the left supralevator space adjacent to the rectosigmoid junction. B) A 1.8 cm hypermetabolic left perirectal lymph node (maximum SUV=7.6). SUV, standardized uptake value

Pelvic MRI revealed a 1.7 cm enhancing lesion in the left supralevator space and diffuse thickening of a 7 cm distal rectal/anorectal segment (Figure 1A). Biopsy confirmed moderately differentiated invasive squamous cell carcinoma with positive margins. Staging CT and 18-F-fluorodeoxyglucose positron emission tomography/computed tomography (18-F-FDG PET/CT) demonstrated a hypermetabolic anorectal mass (standardized uptake value (SUV) 12.1) and a 1.8 cm hypermetabolic perirectal lymph node, without distant disease (Figure 1B).

The patient underwent definitive chemoradiation with 5-fluorouracil and mitomycin-C (Table 1). Follow-up PET/CT more than two years after definitive treatment showed a complete metabolic response. Proctoscopy performed every six to 12 months over the next several years showed no recurrence.

**Table 1:** Descriptive timeline of patient's disease course. SCCA, squamous cell carcinoma of the anus; HAART, highly active antiretroviral therapy; PET/CT, positron emission tomography/computed tomography

Timeline of Disease Course	
Year	Event
2014	Diagnosis of SCCA, HIV-positive, nonadherent to HAART
2014	Definitive chemoradiation (5-FU + mitomycin-C)
2017	PET/CT showed complete metabolic resolution
2017-2023	Regular surveillance, no recurrence
2023	Multiple interruptions in HAART due to psychosocial stress
2025	Pulmonary recurrence, 2.7 cm lung nodule

Metastasis

At age 51, nine years post-treatment, he presented with night sweats, chills, chest pain, abdominal pain, hematochezia, and unintentional weight loss. He was afebrile and hemodynamically stable. Laboratory results showed hemoglobin 10.2 g/dL, CD4 512 cells/mm^3^, and normal LDH and CRP levels.

**Figure 2:**
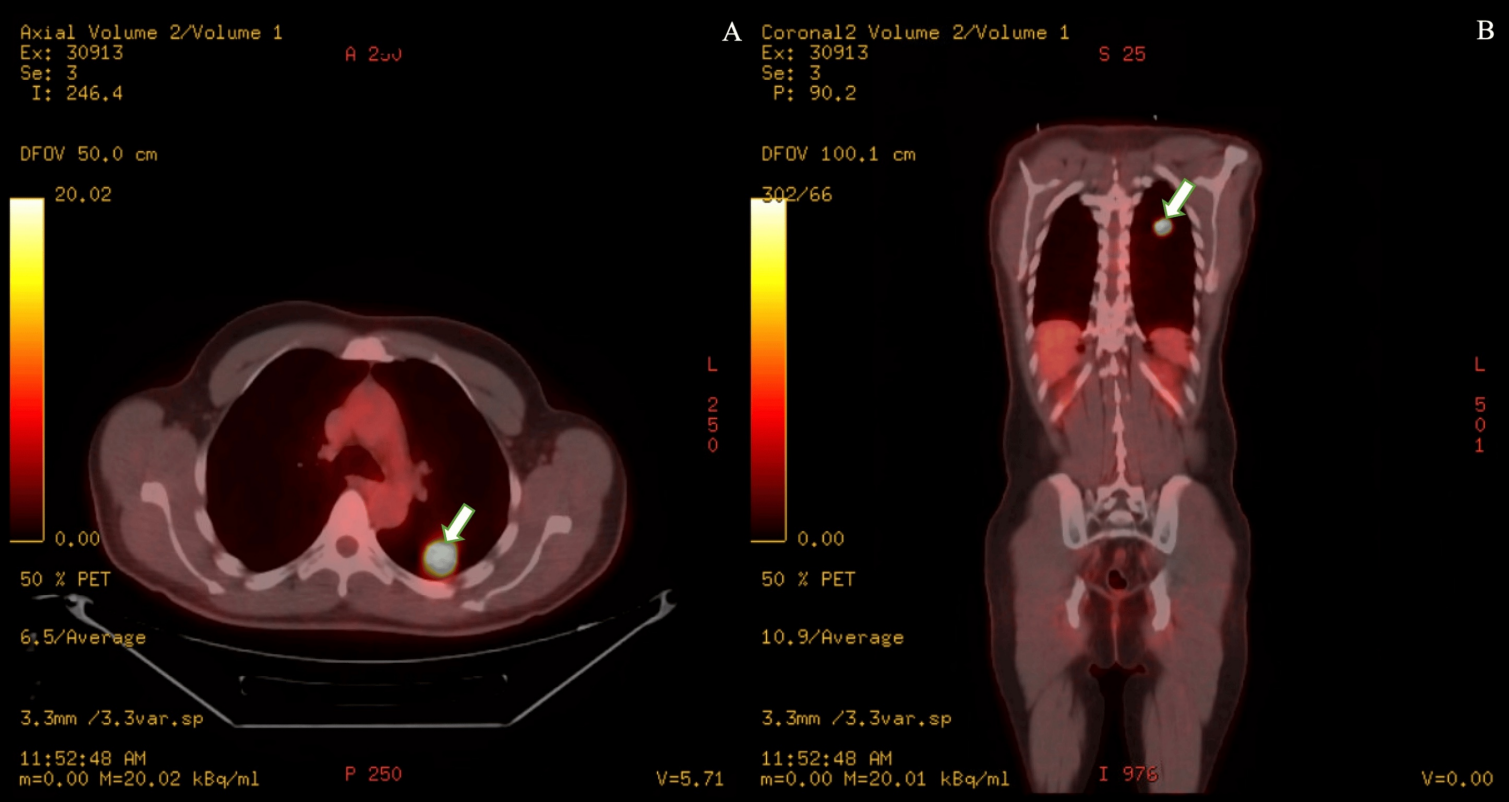
Hyperbolic left lower lobe lung nodule with a maximum SUV of 17.2; A) axial view, B) coronal view. SUV, standardized uptake value

CT angiography revealed a 2.7 cm solid nodule with spiculated borders in the left lower lobe. CT-guided biopsy confirmed squamous cell carcinoma positive for P40 and P16, consistent with HPV-associated disease. 18-F-FDG PET/CT showed intense uptake in the lesion (SUV 17.2), with no other sites of disease (Figure 2). Colonoscopy identified two benign tubular adenomas (resected) and one hyperplastic rectal polyp (resected). No evidence of local recurrence was seen. Tumor markers were unremarkable.

Management

The multidisciplinary review considered surgery. However, surgical resection was deferred because the patient’s HIV-associated immunosuppression, prior pelvic radiation, and comorbidities made systemic therapy preferable. He was started on carboplatin (area under the curve (AUC) 5) and paclitaxel (80 mg/m² on days 1, 8, and 15 of each 28-day cycle) plus retifanlimab (anti-PD-1). Treatment was complicated by immune-mediated hepatitis, requiring discontinuation of immunotherapy. Chemotherapy was resumed after the resolution of liver enzyme abnormalities.

## Discussion

Anal carcinoma most commonly presents with symptoms such as rectal bleeding, anal pain, changes in bowel habits, or the presence of a palpable mass. Accurate diagnosis and effective treatment require a thorough understanding of anal anatomy and lymphatic drainage patterns, and early detection is critical for improving prognostic outcomes. While clinical evaluation and imaging are essential components of the diagnostic workup, anoscopy with biopsy remains the gold standard for obtaining histopathologic confirmation.

Treatment typically involves a multidisciplinary approach, including surgical resection, radiation therapy, and chemotherapy [4]. Metastatic anal cancer at presentation is relatively less common and represents a therapeutic challenge, as this population has a poor prognosis, with five-year overall survival rates estimated at approximately 10% in men and 20% in women [8]. Poor prognostic factors include male sex, age over 65 years, advanced T stage, nodal involvement, and poorly differentiated tumor histology [9]. The standard first-line systemic therapy for metastatic disease remains a combination of cisplatin and 5-fluorouracil. Despite the emergence of several small, non-randomized phase II trials, there has been minimal therapeutic advancement in the management of metastatic SCCA over the past two decades [10].

This report highlights a rather interesting aberration from the usual recurrence timeline of three to five years, without any evidence of locoregional manifestations. It is essential to note that immunosuppressed patients have poor tolerance of chemotherapy and response to chemoradiation, and an increased rate of recurrence, along with unique displays of tumor recurrence [11].

Notably, this patient’s history of intermittent HAART adherence due to psychosocial stressors likely contributed to his sustained immunosuppression, as reflected by his fluctuating antiretroviral regimens and multiple hospitalizations over the years. At the time of metastasis, he had a CD4 count of 512 cells/mm³ yet exhibited symptoms and radiologic findings concerning malignancy. His diagnosis of HPV-positive pulmonary squamous cell carcinoma was confirmed on biopsy, and PET/CT demonstrated no additional sites of disease.

The patient initially opted for aggressive systemic therapy with carboplatin, paclitaxel, and the PD-1 inhibitor retifanlimab. During treatment, he developed immune-related hepatitis, characterized by elevated liver enzymes (AST and ALT >5 × upper limit of normal). Retifanlimab was temporarily discontinued while supportive care, including corticosteroids, was administered. Chemotherapy with carboplatin and paclitaxel was also paused briefly during the acute phase. Once liver function tests normalized, chemotherapy was resumed, while immunotherapy remained on hold until reassessment by the oncology team.

Diagnosis of metastatic anal SCC was confirmed by immunohistochemical staining for P40 and P16, which are instrumental in distinguishing HPV-associated anal SCC metastases from primary pulmonary squamous cell carcinoma. P40, a sensitive marker for squamous differentiation, and P16, a surrogate for oncogenic HPV infection, are typically co-expressed in metastatic anal SCC. In contrast, primary pulmonary SCC often exhibits P40 positivity without corresponding P16 expression. This differential expression pattern aids in accurately identifying the origin of lung lesions [3,12].

This case highlights several important clinical points. First, late metastasis can occur long after standard surveillance windows, particularly in immunocompromised patients. Second, intermittent HAART adherence may contribute to sustained immunosuppression and potentially influence tumor recurrence patterns. Finally, immunohistochemical markers such as P40 and P16 provide critical diagnostic guidance when evaluating pulmonary lesions in patients with a history of HPV-associated SCCA. Clinicians should maintain a high index of suspicion for metastatic disease in similar patients, and long-term surveillance strategies may need to be tailored to account for these risks.

## Conclusions

The interplay between the use of immunosuppressive antiretroviral medications to prevent life-threatening complications of HIV and neoplastic recurrence in HIV-positive cancer survivors is complex and evolving. This case of unusually late metastatic recurrence underscores the need for further research on strategies to reduce relapse risk while supporting sustained HAART adherence. It also serves as a clinical reminder: when evaluating new nodules in immunocompromised patients, clinicians should maintain a high index of suspicion for metastasis, even in the absence of locoregional disease. Early consideration may accelerate diagnosis and improve long-term outcomes.
